# Pembrolizumab-Induced Capillary Leak Syndrome: A Rare but Life-Threatening Immune-Related Adverse Event

**DOI:** 10.7759/cureus.95038

**Published:** 2025-10-21

**Authors:** Roxanne K Branford, Luis Hernandez Lopez, Adriana P Gonzalez Frontera, Margaret Ledea

**Affiliations:** 1 Internal Medicine, Ross University School of Medicine, St. Michael, BRB; 2 Internal Medicine, Cleveland Clinic Florida, Weston, USA; 3 Internal Medicine, University of Medicine and Health Sciences, Basseterre, KNA

**Keywords:** anasarca, capillary leak syndrome, immune checkpoint inhibitors, immunotherapy complications, pembrolizumab toxicity, pleural effusion

## Abstract

Capillary leak syndrome (CLS) is an exceptionally rare and potentially life-threatening adverse event characterized by the abrupt shift of intravascular fluid into the extravascular compartment, leading to profound hypotension, edema, and potential multi-organ dysfunction. This condition has been associated with immune checkpoint inhibitors (ICIs) such as pembrolizumab. We present the case of a 67-year-old woman with a history of squamous cell lung carcinoma who underwent four cycles of pembrolizumab and presented multiple times with progressive shortness of breath, anasarca, and dyspnea on exertion. Clinical evaluation ruled out common causes of respiratory compromise and fluid overload, prompting consideration of immune-mediated pathology. Diagnostic studies and clinical evolution supported a diagnosis of CLS, underscoring the importance of early recognition and intervention. This case highlights CLS as a severe but treatable toxicity of pembrolizumab, emphasizing that timely detection and appropriate supportive care are essential for favorable clinical outcomes.

## Introduction

Capillary leak syndrome (CLS) is a rare condition where fluid leaks from capillaries into surrounding tissues, leading to a rise in hematocrit and potential complications such as edema, shock, organ failure, and cardiovascular collapse [1]. While sepsis is the most common cause, other conditions such as idiopathic systemic capillary leak syndrome (SCLS), engraftment syndrome, ovarian hyperstimulation syndrome, hemophagocytic lymphohistiocytosis, viral hemorrhagic fevers, autoimmune diseases, and certain drugs like monoclonal antibodies can also trigger CLS [2].

Pembrolizumab is a monoclonal antibody that targets the programmed death-1 (PD-1) receptor on T cells. Its mechanism of action involves binding to the PD-1 receptor and blocking its interaction with its ligands, PD-L1 and PD-L2. Normally, engagement of PD-1 by its ligands inhibits T-cell proliferation and cytokine production, leading to reduced immune surveillance and allowing tumor cells to evade immune detection. By blocking this interaction, pembrolizumab releases the PD-1 pathway-mediated inhibition of the immune response, thereby enhancing T-cell-mediated anti-tumor activity. Approved by the Food and Drug Administration (FDA) in 2014 for advanced melanoma, it is now widely used for many other types of cancers [3].

CLS has been documented in conditions such as sepsis, various autoimmune diseases, and hematologic malignancies, but its occurrence in patients receiving immune checkpoint inhibitors (ICIs) such as pembrolizumab remains poorly understood. To date, there are limited reports describing pembrolizumab-induced CLS, making its pathophysiology, risk factors, and optimal treatment strategies uncertain [4,5]. Given its potential for rapid deterioration, increased awareness and early intervention are critical to improving patient outcomes.

## Case presentation

A 67-year-old woman with a history of stage 3A squamous cell lung carcinoma underwent a left upper lobe lobectomy in December 2023, followed by adjuvant chemotherapy and immunotherapy with pembrolizumab from May to December 2024. After completing her immunotherapy regimen, she developed several symptoms, including dyspnea that worsened with exertion, bilateral posterior chest pain when lying supine, and complaints of bilateral pleural effusions, weight gain, bilateral lower extremity edema, ascites, watery eyes, and tinnitus. These symptoms were attributed to pembrolizumab-induced CLS, diagnosed by exclusion.

In January 2025, her oncologist recommended she go to the emergency department for worsening symptoms of dyspnea. She underwent bilateral thoracentesis, with 1,800 cc removed from the right side and 1,100 cc from the left. Fluid analysis revealed negative results for anaerobic and aerobic bacterial and fungal cultures, and cytology was negative for malignancy. While her symptoms initially improved, they worsened again. In February 2025, she presented to the emergency department with anasarca, dyspnea on mild exertion, generalized swelling (especially bilateral leg edema), left-sided forefoot nerve pain, orthopnea, abdominal distention, early satiety, and a 15-pound weight gain since her last admission. This was her third emergency visit for similar symptoms, which persisted despite previous interventions such as diuresis, bilateral thoracentesis, and high-dose intravenous methylprednisolone (125 mg/2 mL).

**Figure 1:**
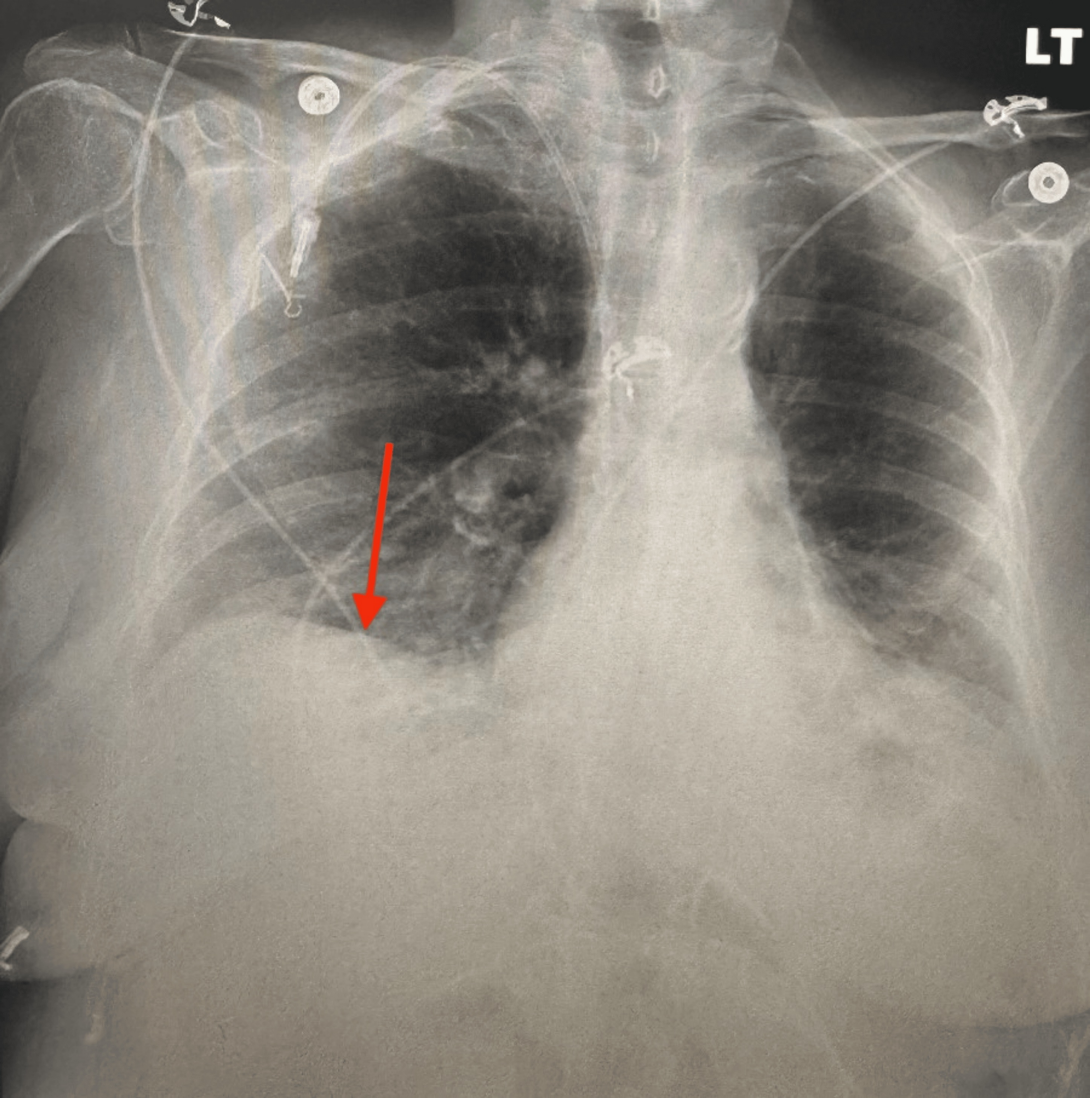
Chest X-ray on admission showing bilateral pleural effusions, more prominent on the right (red arrow)

The patient's vital signs were remarkable for hypotension. On examination, she had hypervolemia, diminished breath sounds at the bilateral lung bases, dullness to percussion, abdominal distention, moderate (2+) bilateral lower extremity edema, and persistent bilateral subconjunctival edema. Laboratory results showed leukocytosis (white blood cell count: 13.46 × 10^3^/µL), aspartate aminotransferase of 13 U/L, alanine aminotransferase of 141 U/L, mildly elevated N-terminal pro-B-type natriuretic peptide (NT-proBNP) (150 pg/mL), low hemoglobin, and a baseline albumin of 2.9 g/dL (Table 1) [6]. Imaging showed bilateral pleural effusions, larger on the right side (Figure 1), low probability of pulmonary embolism on ventilation/perfusion scan, cirrhotic liver morphology (likely from congestive hepatopathy) on right upper quadrant ultrasound, and an unremarkable echocardiogram.

**Table 1:** Key laboratory findings WBC: white blood cell, NT-proBNP: N-terminal pro-B-type natriuretic peptide, AST: aspartate aminotransferase, ALT: alanine transaminase

Laboratories	Normal range	Patient value
WBC	4,500-11,000 × 10^9^/L	13.46 × 10³/µL
AST	12-38 U/L	13 U/L
ALT	10-40 U/L	141 U/L
NT-proBNP	<125 pg/mL	150 pg/mL
Albumin	3.5-5.5 g/dL	2.9 g/dL

**Figure 2:**
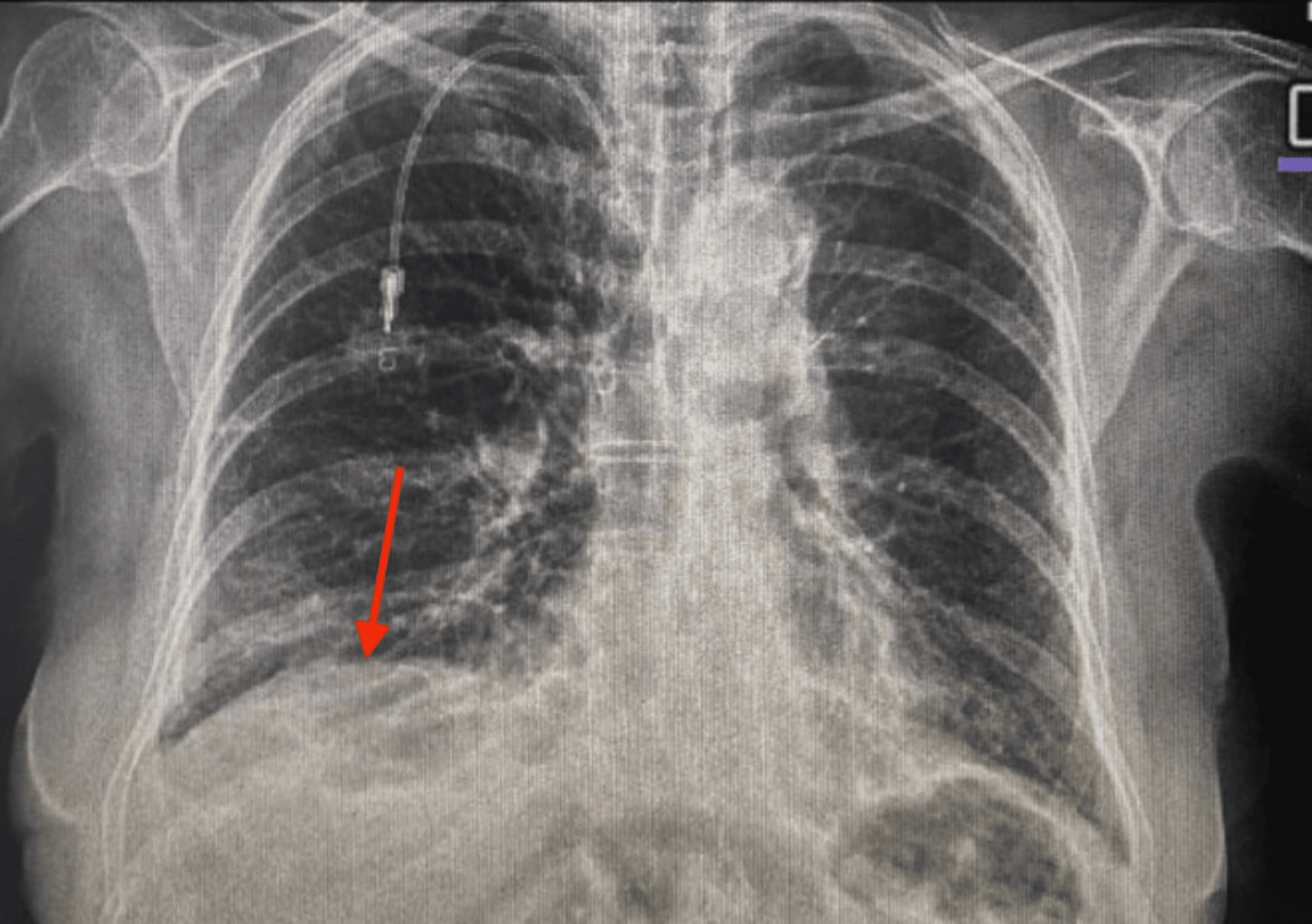
Chest X-ray following thoracentesis demonstrating residual right-sided effusion (red arrow) with interval bilateral improvement

A pulmonology consultant recommended intravenous immunoglobulin (IVIG), furosemide, and methylprednisolone adjusted from 125 mg to 60 mg to optimize intravascular volume and facilitate fluid mobilization. The patient underwent right and left thoracentesis on February 26 and 27, respectively, which showed reduced pleural effusions on X-rays (Figure 2) and improved symptoms. At discharge in March 2025, she transitioned from intravenous methylprednisolone to oral prednisone (60 mg) and was advised to continue weekly IVIG outpatient, as her anasarca and respiratory symptoms were improving.

## Discussion

Systemic capillary leak syndrome (SCLS) is a rare and under-recognized disorder characterized by recurrent episodes of plasma extravasation leading to hypovolemia, edema, and organ dysfunction [7,8]. Reviews of the syndrome describe both idiopathic and secondary forms, with the latter linked to hematologic malignancies, infections, and drug exposures [8,9]. Large registry studies, such as the European series of 28 patients, highlight its chronic relapsing nature and significant morbidity [10]. Although rare, authoritative definitions such as those from the US National Library of Medicine emphasize its distinct clinical trial of hypotension, hemoconcentration, and hypoalbuminemia [7,9], making differentiation from other causes of fluid overload critical.

Classical SCLS is most often associated with monoclonal gammopathy, hematologic malignancies, or elevated pro-inflammatory cytokines such as interleukin-2 (IL-2) and tumor necrosis factor-alpha (TNF-α) [1,2,11]. Narrative reviews highlight that classical Clarkson disease is typically marked by hypotension, hemoconcentration, and hypoalbuminemia [12]. In contrast, immune checkpoint inhibitor (ICI)-associated CLS is far less common and appears to involve a distinct immunopathological mechanism. Inhibition of the PD-1 pathway by pembrolizumab potentiates T-cell activity, which may in turn promote endothelial damage and vascular permeability [3,8,13]. This differs from classical SCLS, where vascular permeability is more directly mediated by cytokine storms and endothelial apoptosis [1,2,9].

Pembrolizumab-induced CLS represents a rare but potentially fatal immune-related adverse event (irAE) requiring prompt recognition and targeted management. The diagnostic challenges presented in this case, including recurrent fluid overload, pleural effusions, and respiratory compromise in a patient with squamous cell lung carcinoma, are consistent with previously reported cases of ICI-associated CLS, albeit limited in number [5,13]. In our case, the absence of paraproteinemia, hypotension, or hemoconcentration hallmarks of Clarkson disease supports a mechanism distinct from cytokine-driven vascular injury, aligning more closely with prior ICI-CLS reports [5,13].

A key differentiator in ICI-induced CLS is the absence of paraproteinemia or cytokine-driven inflammation, commonly seen in idiopathic or sepsis-related CLS [1,2,9]. Similar observations were reported by Qin et al., who documented pembrolizumab-induced CLS with lymphatic dysfunction responsive to corticosteroids and IVIG [5], while other case reports and registry reviews further support immune-mediated endothelial injury without monoclonal protein involvement [10,13]. Compared to those patients, ours exhibited slower resolution, likely due to delayed steroid initiation and possible greater endothelial injury.

The patient’s transient response to diuretics and persistent anasarca further distinguishes ICI-induced CLS from congestive causes of fluid overload. This clinical pattern suggests that fluid accumulation in ICI-CLS is often resistant to conventional diuretic therapy and instead requires immunosuppressive treatment to address underlying endothelial inflammation [5,13].

The cornerstone of treatment in severe irAEs remains corticosteroids. Our management approach of initiating high-dose methylprednisolone followed by a tapering regimen is supported by guideline-based recommendations from the American Society of Clinical Oncology (ASCO) [9] and the National Comprehensive Cancer Network (NCCN) [11]. This parallels prior ICI-CLS cases where early corticosteroid use led to quicker stabilization [5,13], although our patient’s slower recovery underscores the importance of timely initiation. In steroid-refractory cases, escalation to IVIG has been reported with success [13]. The adjunctive use of albumin with loop diuretics, as seen in our case, has been described in sepsis-related and idiopathic CLS [2,9,10], although its utility in ICI-induced CLS remains anecdotal and warrants systematic evaluation.

While literature on pembrolizumab-induced CLS remains sparse, the growing use of ICIs across various malignancies calls for heightened awareness of such complications. Pharmacovigilance analyses and systematic reviews suggest that ICI-related CLS may be under-recognized, highlighting the need for mechanistic and biomarker research [10,13].

This case reinforces the critical need for differential diagnosis in patients receiving ICIs who present with unexplained edema or respiratory compromise. Given the pathophysiological differences between classical and ICI-induced CLS, future research should focus on identifying predictive biomarkers (e.g., vascular endothelial growth factor (VEGF), angiopoietin-2 (Ang-2), or intercellular adhesion molecule-1 (ICAM-1)), clarifying immune mechanisms behind endothelial injury, and establishing evidence-based treatment algorithms. Until such data are available, clinicians must adopt a multidisciplinary and high-suspicion approach to manage these complex cases effectively.

## Conclusions

This case highlights the challenges faced in managing a patient with pembrolizumab-induced CLS, compounded by recurrent pleural effusions, ascites, and generalized edema. Clinicians should maintain a high level of suspicion for CLS in patients receiving immune checkpoint inhibitors, particularly those presenting with fluid overload and pleural effusions. Prompt, aggressive management is crucial to prevent complications such as shock, organ dysfunction, and mortality. Multidisciplinary management, including IVIG, albumin, diuretics, and steroid therapy, is essential in optimizing outcomes. Further research should focus on prospective studies to determine the frequency and duration of IVIG administration needed to prevent CLS exacerbation in patients on pembrolizumab therapy.
